# Abstracts in high profile journals often fail to report harm

**DOI:** 10.1186/1471-2288-8-14

**Published:** 2008-03-27

**Authors:** Enrique Bernal-Delgado, Elliot S Fisher

**Affiliations:** 1 Health Services Research Unit, Institute for Health Sciences in Aragon, Gómez Laguna Av. 25, 50009 Zaragoza, Spain; 2 Center for Healthcare Research and Reform, Dartmouth Institute for Health Policy and Clinical Practice Lebanon, 03766 NH, USA

## Abstract

**Background:**

To describe how frequently harm is reported in the abstract of high impact factor medical journals.

**Methods:**

*Design and population*: We carried out a blinded structured review of a random sample of 363 Randomised Controlled Trials (RCTs) carried out on human beings, and published in high impact factor medical journals in 2003. *Main endpoint*: 1) Proportion of articles reporting harm in the abstract; and 2) Proportion of articles that reported harm in the abstract when harm was reported in the main body of the article. *Analysis*: Corrected Prevalence Ratio (cPR) and its exact confidence interval were calculated. Non-conditional logistic regression was used.

**Results:**

363 articles and 407 possible comparisons were studied. Overall, harm was reported in 135 abstracts [37.2% (CI95%:32.2 to 42.4)]. Harm was reported in the main text of 243 articles [66.9% (CI95%: 61.8 to 71.8)] and was statistically significant in 54 articles [14.9% (CI95%: 11.4 to 19.0)]. Among the 243 articles that mentioned harm in the text, 130 articles [53.5% (CI95% 47.0 to 59.9)] reported harm in the abstract; a figure that rose to 75.9% (CI95%: 62.4 to 86.5) when the harm reported in the text was statistically significant. Harm in the abstract was more likely to be reported when statistically significant harm was reported in the main body of the article [cPR = 1.70 (CI95% 1.47 to 1.92)] and when drug companies (not public institutions) funded the RCTs [cPR = 1.29 (CI95% 1.03 to 1.67)].

**Conclusion:**

Abstracts published in high impact factor medical journals underreport harm, even when harm is reported in the main body of the article.

## Background

Adherence to the best clinical evidence has become an important guiding principle in both the medical and health policy decision-making processes. However, concern is growing about the way evidence, and particularly harm, is presented to doctors and policy makers particularly because benefit has the propensity to be reported more frequently than harm [1-3], especially when conflicts of interest are present (e.g. when drug companies fund research) [4-7]. Recent research has shown how trial reports fail to either define or record adverse events [8,9] or only partially report them [10].

Because of its relevance in medical decision making [11-17], quality of reporting abstracts, and particularly the way harm is reported, has been considered an important issue [18-20]. In fact, besides its generic recommendation in 2004 [21], CONSORT initiative has recently published a new statement to better report journal and conference abstracts, including a specific reference to harm [22,23].

Little is known, however, about the way abstracts report harm in phase III and IV randomized controlled trials published in high impact journals, those considered gold standard for clinical evidence and therefore, those with the highest capacity to influence medical decision making. Our objective was to describe how frequently harm is reported in the abstracts of high impact factor medical journals.

## Methods

### Design

A blinded structured review of abstracts reporting original clinical research on human beings published in high impact factor medical journals was carried out.

### Population and setting

**Figure 1:**
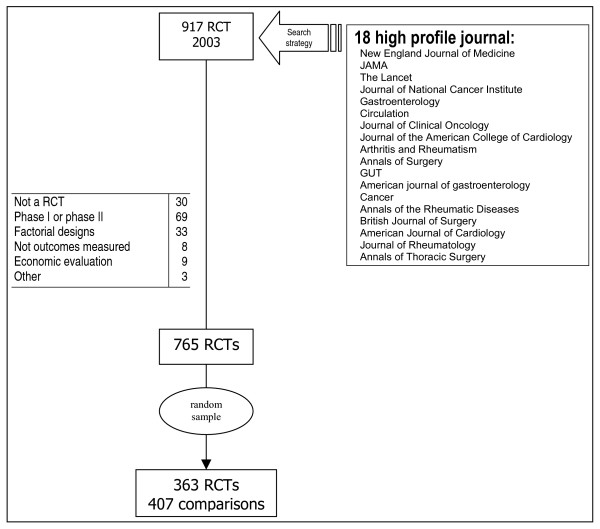
Sample selection flow diagram.

A purposive sample of journals was selected based on their 2003 impact factor (Figure 1). *Excluded from the sample were*: Phase I and phase II RCTs, factorial designs, economic evaluations and RCTs assessing process measures (instead of health outcome endpoints). Those RCTs fulfilling inclusion criteria were sorted by their PMID number. A consecutive id number from 1 to 363 was assigned. Then, a random numbers table was used to recover the sample of 2003 RCTs to be studied (See Additional file 1). A PubMed search strategy was used to retrieve RCTs (Table 1).

**Table 1:** RCT search strategy

#1 randomized controlled trial. pt
#2 limit #1 to (clinical trial, phase i or clinical trial, phase ii)
#3 #1 not #2
#4 limit #3 to yr = 2003
#5 limit #4 to human
#6 limit #5 to journal article
#7 comment. pt
#8 #6 not #7
#9 #8 not letter. pt

### Main endpoint

Two main endpoints were examined in this work: 1) the proportion of RCTs reporting or quantifying harm in the abstract; and 2) the proportion of RCTs that mention or quantify harm in the abstract when harm was reported in the main body of the article. Harm was defined as any possible adverse consequence of an intervention or therapy [21].

### Other studied predictors

We considered as explanatory factors: a) funding source (drug or device companies vs public institutions); b) use of placebo as control group; c) the sample size (using the median value, 200 patients, as a threshold); d) the main endpoint direction of effect (defined as favouring, being neutral or opposing the intervention); e) clinical significance of harm in the text (using mortality vs no mortality as a *proxy*); and f) whether the harm reported in the main text was statistically significant (yes vs no).

### Data abstraction

An instrument was developed *ad hoc *to retrieve key information from each article. Its construct and face validity was assessed by two independent researchers, blinded to the study hypothesis and the RCTs authors and journals.

Once the tool was designed, information from each article was obtained following a three steps method. In the first step, one of us (EBD) selected the articles regarding the inclusion criteria and entered the information about topic, treatment groups, and number of treatment arms. Secondly, two trained junior researchers, blinded to the hypothesis of the study received an electronic copy of each article – any single reference to the authors and to the journal was masked- and retrieved all the remaining information using the developed tool. In order to control inter-observer reliability, accuracy between observers was evaluated before (pilot study) and during the research period. Accuracy ranked from 73% to 82% in main variables. Disagreement was resolved by a third blinded observer using consensus when necessary.

### Analysis

Descriptive measures and Exact confidence intervals with α-error equalling 5% were calculated. In order to assess the effect of predictors over the main endpoints, bivariate non-conditional logistic regression was used. Due to the high prevalence of the outcome of interest a Corrected Prevalence Ratio (cPR) was applied using the method suggested by Zhang [24,25]. STATA/SE version 8.0 was used to perform analysis.

## Results

765 RCTs met the study inclusion criteria. A random sample of 363 articles was selected for review. Since 42 articles had more than one treatment arm, 407 possible comparisons were considered. (Figure 1 details exclusions).

The vast majority of RCTs studied therapeutic interventions (89%) and signs or symptoms were considered the main endpoint in over 60% of studies. 52% of articles used placebo as a control group, 51% of articles had fewer than 200 patients (total sample size) and the plurality of articles favoured intervention arm (41%). Of note, 25% of abstracts reported no quantitative information on the impact of the intervention on the primary study endpoint.

Harm was reported or quantified in 135 abstracts [37.2% (CI95%:32.2% to 42.38%)], 40% of them (54 out of 135) used some kind of numerical data: 33 abstracts reported either "p values" (27 articles) or confident intervals (6 articles). The remaining 21 abstracts referred to harm in terms of percentage (or mean) of events in each arm, though no statistical information was offered. When no numerical data was reported (81 articles) unspecific expressions like *"there were no differences in adverse events" *were used. In addition, harm was reported in the main text of 243 articles [66.9% (CI95%: 61.8 to 71.9)] and was statistically significant in 54 articles [14.9% (CI95%: 11.4 to 19.0)].

**Figure 2:**
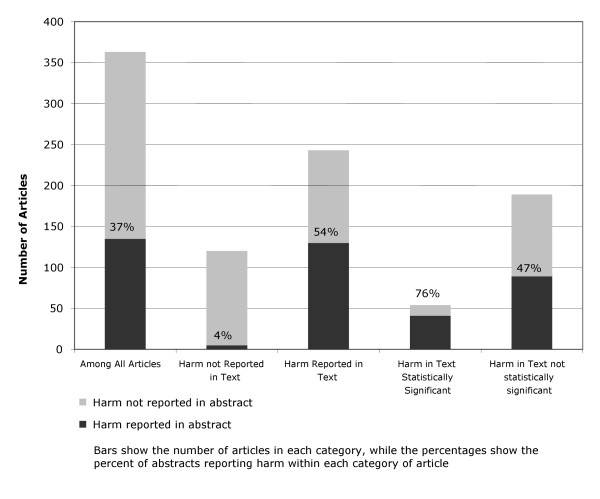
Proportion of randomized controlled trials in high profile medical journals reporting harm in abstracts.

Among the articles that reported harm in the text, 130 articles [53.5% (CI95% 47.0% to 59.9%)] reported harm in the abstract. 41 articles [75.9% (CI95%:62.4% to 86.5%)] reported harm in the abstract when harm in the text was reported as statistically significant (Additional details are shown in Figure 2). When the 407 different comparisons (instead of studies) were analysed, figures were similar.

Table 2 shows results from bivariate analyses. A very strong relationship between reporting harm in the text and in the abstract (cPR = 8.71) was found; however, this relationship was not so important when either statistical significance (cPR = 1.7) or clinical relevance were considered (cPR = 1.43). On the other hand, where funding sources were concerned, harm in the abstract was less likely to be reported when public institutions (as opposed to companies) funded the RCTs (cPR = 0.77). The remaining factors did not show a statistically significant relationship with the probability of reporting harm in the abstract.

**Table 2:** Factors influencing harm reported in the abstract

	**cPR (CI95%)**
Funding*	
drug or device companies	1
public institutions	0.77 (0.60 to 0.97)
**Placebo**	
other control	1
placebo	0.97 (0.81 to 1.13)
**Sample size**	
less than 20	1
equals or more than 200	1.14 (0.96 to 1.33)
**Direction of effect**	
beneficial to intervention	1
neutral or against the intervention	1.00 (0.83 to 1.19)
**Harm in text**	
no harm in text	1
harm in text	8.71 (5.90 to 12.01)
**Clinical significance of harm**	
no mortality nor composite in text	1
mortality or composite in text	1.43 (1.22 to 1.65)
**Harm in text statistically significant**	
not statistically significant	1
statistically significant	1.70 (1.47 to 1.92)

cPR: Prevalence Ratio estimated by logistic regression, and corrected by applying the method proposed by Zhang [24]; CI: Confidence Intervals with error type I equals 5%; *363 articles except for funding in which sample size was n = 226

When logistic regression was applied to the articles which reported harm in the text, funding source and reporting mortality in the body of the article did not remain in the model. Only reporting statistically significant harm in the text was related to the probability of reporting harm in the abstract [cPR = 1.25 (CI95% 1.12 to 1.38)].

## Discussion

We have found that 33% of the articles in our sample did not report harm in the text; additionally, 46.5% of the studies that documented harm in the body of the article failed to report these harm in the abstract. Unfortunately, although different approaches to the study of abstracts have been developed, there is no similar research with which compare the coherence and consistency of these results.

With regard to the underlying factors of these results, we have found that the probability of harm being reported in the abstract depends on harm reporting in the body of the article, particularly, when statistically significant harm occurs. This finding is consistent with previous evidence in which researchers themselves suggest that the lack of statistical significance is a potential cause of failure in reporting harm [10]. In the same article, however, authors suggest that another important factor must be considered: clinical significance of harm. Some may consider that we have overlooked a specific assessment of this factor. Our study has certainly not assessed clinical significance itself but we have used a proxy *"reporting mortality in the text as an adverse event"*, which was moderately associated. However, "clinical significance of harm" did not remain in logistic models when statistically significant effects were considered. This may suggest that, in our sample, reporting harm in abstracts is driven more by statistics than by clinical significance of harm.

One other result deserves further attention. As we mentioned above, the vast majority of research has demonstrated that articles funded by industry tend to favour the intervention group; however, when a study was funded by industry harm being reported in the abstract was favoured, even though phase IV RCTs were included in the sample. Some explanations should be argued. On the one hand, studies funded by commercial sources have a larger sample size than those funded by public institutions and, therefore, they have more statistical power to detect adverse events if they exist. In actual fact, while the mean sample size of RCTs sponsored by industry reached 1619 patients, mean sample size of publicly funded RCTs counted for 793 patients. Certainly, when we controlled for study size, the effect of funding was no longer significant. Another alternative explanation is related to the strict control that industry faces when developing a new drug. Industry is required by National Agencies to collect and report efficacy and safety. This requirement contrasts with a more relaxed policy in the case of public funded RCTs in which authors are only asked to declare their own conflict of interests.

### Limitations

Some limitations of our study must be reported. Firstly, as a purposive sample of journals, our results must only be referred to the journals we have studied. In any case, we have selected high profile literature (in terms of impact factor and professional acknowledgement) under the hypothesis that these journals should accomplish high standards of reporting more frequently than others. If this hypothesis is true, worse results should be expected in the remaining literature but our data is not able to state this. Secondly, it could be argued that factors exist which were not considered in this study. Even though this could be true, the vast majority of variation in reporting harm in the abstract was explained by the presence of harm in the body of the article (ROC curve area equals 0.73 when logistic models were fitted). Therefore, the potential effect of other factors would be residual in any case. Finally, due to the fact that we used an instrument to retrieve the information from the RCTs, and two independent blinded reviewers were involved, misclassification could be argued as a possible source of bias. Even though bias is possible, agreement between both reviewers was assessed before consensus and they reached an accuracy index over 75% in the main variables.

### Implications

Abstracts are the most widely read summaries of research findings and are an important source of information for clinicians and policy makers; particularly abstracts of phase III&IV RCTs published in high profile journals. Additionally, the electronic abstract of almost all published articles can easily be obtained from electronic databases. Both elements combined with the fact that a third of the articles in our sample did not report harm and more than half the studies that documented harm in the body of the article failed to report this harm in the abstract strongly support the reporting policies suggested by the two latest CONSORT statements; particularly in those aspects related to appropriate database indexing and information retrieval [21] and reporting important adverse (or unexpected) effects of an intervention [22].

However, our results suggest a new perspective to the statement because the probability of reporting harm in the abstract is mainly driven by the statistical significance rather than the "clinical relevance" of the finding. Probably, a more specific statement will be needed in order to determine what "important adverse (or unexpected) effects of an intervention" mean for the statement: whether it be statistical significance (which is influenced by the statistical power of the RCTs to detect harm), or clinical importance (which entails a more difficult definition).

Finally, our study suggests that to better reach CONSORT objectives, a new rationale of reporting harm should be adopted, resting on the need for clinicians and policy makers to understand that almost all interventions have both benefits and harm.

## Conclusion

In conclusion, abstracts published in high impact factor medical journals underreport harm even when the articles provide information in the main body of the article. The results should encourage researchers, public funding institutions and editors to pay more attention to the way benefits and harm are reported.

## Competing interests

The author(s) declare that they have no competing interests.

## Authors' contributions

EBD, the guarantor of the study, had full access to all the data, and takes responsibility for the integrity of said data and the accuracy of the analysis. EBD contributed to the study conception and design, acquisition of data, analysis and interpretation of results, and drafting the article. EF contributed to the conception and design of the study, and drafting the article.

## Pre-publication history

The pre-publication history for this paper can be accessed here:



## Supplementary Material

Additional file 1 The file contains title, authors, source and abstract of the RCTs included in the study. Click here for file
